# Evaluation of the Yes to Veg! Programme, a Food Systems Approach to Increase Vegetable Exposure and Agency in Pre‐School Age Children: A Quasi‐Experimental Study

**DOI:** 10.1111/mcn.70145

**Published:** 2025-12-04

**Authors:** Ada Lizbeth Garcia, Zabrina Zerr, Irina Martin, Alison Parrett

**Affiliations:** ^1^ Human Nutrition, School of Medicine, Dentistry and Nursing, College of Medical, Veterinary & Life Sciences University of Glasgow Glasgow UK; ^2^ Nourish Scotland Edinburgh UK

**Keywords:** child agency, early years, food systems, pre‐school children, vegetable intake

## Abstract

Children's early years food environment can influence dietary habits. We evaluated Yes to Veg! a 4‐week nursery‐based programme on pre‐school children's vegetable exposure, consumption and agency. A quasi‐experimental study in 11 nurseries (6 intervention/5 controls) located in socio‐economically deprived areas of Glasgow, Scotland. Yes to Veg! delivered locally grown fresh vegetables once‐per‐week for children's daily nursery activities. Control nurseries received standard healthy eating recommendations. Parental pre‐ and post‐questionnaires measured child vegetable exposure (vegetables tried from a 27‐item list), consumption frequency (1 = once‐per‐week to 5 = everyday) and variety consumed (0 = none/1 = 1‐4/2 = 5‐9/3 = 10+ kinds). Qualitative comments reported by parents, nursery staff and from researcher observations were extracted for qualitative themes. From 257 parent‐child dyads recruited, 57 (*n* = 34 intervention/*n* = 23 control, child mean age 51 months) completed both questionnaires. Vegetables tried [Mean(SD)] did not change between intervention [total score pre 16.7(4.5) vs 16.8(5.6) post, difference 0.19(0.6), *p* = 0.765] and control group [total score pre 16.4(5.3) vs 16.0(5.6) post, difference −0.39(0.57), *p* = 0.503]. Median pre‐ and post‐consumption frequency in both groups was 4 (most days); the variety of vegetables consumed was higher in intervention (5‐9 items) vs control (1‐4 items) and these measurements didn't change between pre‐ and post. Vegetable agency increased in the intervention; parents said children talked more about vegetables at home (91% vs 65% control) and were willing to try vegetables at home (41% vs 34% control); emerging qualitative themes included children's engagement with vegetables, sensory interaction and programme acceptance. Yes to Veg! facilitated exposure, engagement and familiarisation to vegetables, was well implemented and received, but did not change consumption.

## Summary

- A food system approach for the promotion of vegetable consumption in early years involving pre‐school children, parents, nursery staff and local vegetable producers is a promising concept for the promotion of vegetable intake.
- Yes to Veg! facilitated access to vegetables in socio‐economically deprived nurseries and supported children's familiarisation with vegetables.
- Children gain vegetable agency by having direct access to vegetables in the nursery setting.

## Graphical abstract

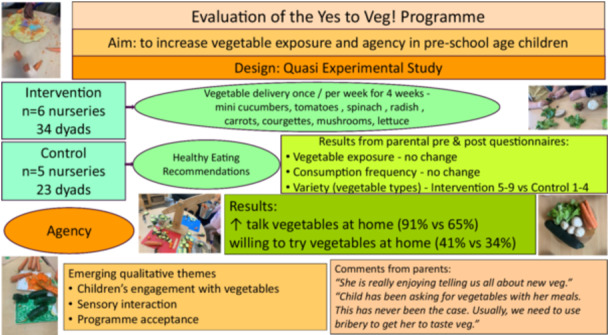

## 1 Introduction

Children's diets in the UK do not meet current recommendations for free sugars, fish, dietary fibre and fruit and vegetables (F&V) (Office for Health Improvement and Disparities 2025). Socioeconomic deprivation is a strong predictor for not meeting F&V recommendations. In Scotland, children living in more deprived households consume less fresh fruit (75 g/day) and vegetables (95 g/day) compared to those from more affluent families (103 g fresh fruit/day, 144 g vegetables/day) (Jaacks et al. 2025) and overall show poorer diet behaviours. Diet inequalities reflect health inequalities from an early age, for instance the prevalence of obesity in Scottish children aged 5 years at primary school entry level who live in more deprived households was 15.7% compared to 7.3% in more affluent (Public Health Scotland 2022). This is concerning because early childhood nutrition is essential for optimal growth and development but also because during early years, children acquire food preferences, establish eating habits (Birch and Doub 2014) and develop awareness of their social and physical environment (Brownell et al. 2013). Therefore, interventions to address unhealthy eating behaviours in children living in areas of socioeconomic deprivation should be a priority.

A general area of concern in early years settings is the refusal to consume vegetables (Hendrie et al. 2017). During toddlerhood food fussiness and neophobia are common and vegetables are often reported to be disliked (Holley et al. 2018). In the UK, the median F&V intake in children aged 1.5 to 3 is 152 g/day, well below the 400 g/day recommendations, furthermore children eat more fruit (median 93 g/day) than vegetables (median 63 g/day) (Public Health England 2020). Thus, it is important to develop strategies to promote vegetable consumption during early years as preference for vegetables can be shaped during toddlerhood (Birch and Doub 2014) and can track into later childhood (Fletcher et al. 2017).

Barriers to eating F&V in childhood are multifactorial. Young children's eating behaviours are influenced by parents and carers (Edwards et al. 2022), their surrounding environment and intrinsic traits such as refusal for bitter taste and food neophobia (Issanchou 2017). Developing interventions to address these barriers needs a food system approach that includes a place‐based environmental angle (Hawkes et al. 2015). Early years settings, including day care centres and nurseries, are an important component of the early year's food system and therefore a promising environment to intervene. Our previous research in nursery‐based settings has shown that a parent and child intervention based on sensory learning (sight, touch, smell, taste), repetition, food preparation and play was effective in reducing food fussiness and increasing willingness to try and eat green/bitter vegetables (Garcia et al. 2020). Familiarisation is key in early years' behaviour learning; for example, using books to familiarise pre‐school age children with target green vegetables through “farm to fork visuals” followed by taste and familiarisation by direct delivery of vegetables at home was effective in increasing children's liking for and tasting of green vegetables (Owen et al. 2018). Changing the physical environment can positively contribute to re‐enforcing healthier behaviour, for example modifying access to water in nurseries reduced intake of sugar sweetened juice in pre‐school children (Pinket et al. 2016). “Agency” as described in social cognitive theory is driven by interactive, coordinative, and synergistic dynamics which in the nursery context can play an important role in the acquisition of food related agency (Bandura 2006). Studies in adolescents have described the relevance of agency in food choice (Green et al. 2021) but the role of agency in early years settings is limited. We hypothesised that making changes in the food environment in nursery settings will facilitate agency for behaviour change to improve vegetable consumption. This could be achieved by physically accessing fresh vegetables that can be used in the daily routine of children attending nursery. This could be mediated by encouraging toddlers to play an active role in a food environment that enables vegetable intake with activities such as sensory learning, repetition, free playing, growing, and connecting to local, organic seasonal produce.

“Yes to Veg!” is a holistic approach to facilitate vegetable consumption in pre‐school age children by reducing barriers to intake. The programme includes key people in a child's daily food environment: families and nursery staff, as well as local, seasonal, organic vegetable suppliers. The approach is to use play and senses to facilitate experiential learning with focus on repeated exposure to vegetables' texture and flavour (Issanchou 2017). The aim of this study is to evaluate the Yes to Veg! Programme. We will evaluate the process and short‐term impact on vegetable exposure, consumption and agency.

## 2 Methods

### 2.1 Study Design

This was a quasi‐experimental study. The intervention was the Yes to Veg! Programme and the control received standard healthy eating information. We used pre‐ and post‐questionnaires for quantitative outcomes and researcher observations combined with parental and staff reports for qualitative outcomes and for process evaluation.

### 2.2 Participant Recruitment

Participants were parents of children enroled in the nurseries and nursery staff. For access to nurseries and recruitment we partnered with a local organisation, Nourish Scotland, a charity working to improve the food environment, who developed the Yes to Veg! Programme, and the National Health Service (NHS) Greater Glasgow and Clyde Health Improvement team who work on nutrition promotion activities in council‐run nurseries located in socioeconomically deprived areas of Glasgow. The NHS Health Improvement team reached out to potential nurseries to gauge interest on running the Yes to Veg! Programme. A total of 12 nurseries expressed written interest and they were selected to take part in the study. The allocation to intervention was done on a first‐come first‐served basis. Recruitment and information packs for the 775 potential participants in the 12 nurseries were delivered to the nurseries before the start of the study. The packs were given to parents who had children aged 3‐5 years enroled in the nursery. All packs for parents contained a participant information sheet, a privacy notice, and a consent form and a pre‐questionnaire, in addition the packs for the control group contain a print of the Eatwell Guide, a UK tool for the promotion of healthy eating (Public Health England 2018). The packs for nurseries contained a participant information sheet, a consent form for the teacher responsible for liaising with the researchers, a privacy notice, a form asking what activities they did with vegetables before intervention and post intervention and a poster to advertise the study in the nursery. For the intervention nurseries the pack also contained a list of suggested activities nursery staff could implement during the intervention.

### 2.3 Intervention and Control

The Yes to Veg! Programme's (intervention) aim was to facilitate vegetable consumption in pre‐school age children by reducing barriers to intake by using a food system approach which included (1) key people in a child's daily food environment: families, nursery staff and peers, (2) the supplier of local, seasonal, organic vegetables who came to the nursery once a week to deliver the fresh produce and (3) experiential learning by using senses, play, repeated exposure to vegetables' texture and flavour (Issanchou 2017); the experiential learning which is underpinned by repetition (Caton et al. 2013) was hypothesised to be conducive to agency. The Yes to Veg! Programme consisted of 4 weeks of vegetable deliveries to the participating nurseries. At the start of the week a local supplier delivered 2 types of vegetables per week, one familiar and one less familiar. The amount of vegetables delivered was calculated for all children to be able to use them for the full week and be able to get familiar and comfortable with the vegetables in the nursery environment. This was done using the existing register of children in the nursery. This meant all children attending the nurseries receiving Yes to Veg! had access to vegetables independent of having signed consent for participating in the study. The types and amounts of vegetables (portion per child per week) delivered to the Yes to Veg! nurseries were: week 1, mini cucumbers (1 unit), Heirloom tomatoes (50 g), classic vine tomatoes (50 g); week 2, spinach (100 g), radish (1/3 bunch); week 3, carrots (250 g), courgettes (250 g); week 4: white mushrooms (80 g) and lettuce leaves (80 g). At the start of the intervention nurseries received information with suggestions for what they could do with the fresh produce (Supporting material 1). Suggested activities were that children introduce the interaction with vegetables in their daily routine, this included activities such as welcome the vegetable delivery. Nurseries were also encouraged to create “market stalls” for children to play but also for parents to be encouraged to take home vegetables at the end of the week. Other suggestions were to use the vegetables in activities already occurring in the nursery that could aid child's sensory learning (touch/texture, smell, sight/colour identification, sound and taste; other learning opportunities to engage with numbers could be counting and measuring). Further activities were those related to growing and the environment. An important aspect was to promote free/imaginative play to support agency which included selling, cooking, cutting, or using mud kitchens if they existed in the nursery.

The control group received general healthy eating information only (Eat Well Guide); they were placed on a waiting list to receive the Yes to Veg! Programme later in the year.

### 2.4 Evaluation Outcomes

#### 2.4.1 Quantitative Outcomes

Vegetable exposure and intake were recorded using parental pre‐ and post‐questionnaires adapted from our previous study in a similar population and setting (Garcia et al. 2020). The questionnaires included questions on sociodemographic information (child's gender and age, child's additional support needs, language spoken at home, parental age and household postcode; postcodes were transformed into a digit from 1 (more deprived) to 5 (least deprived) using the Scottish Index of Multiple Deprivation (SIMD) (Scottish Index of Multiple Deprivation SIMD 2012) and questions related to vegetables. To measure vegetable exposure parents were asked what vegetables children had tried before and after the intervention from a list of 27 vegetables. To measure vegetable consumption parents were asked to report children's frequency of vegetable consumption, excluding potatoes, using a scale from 1 to 5 (1 = rarely/never, 2 = once a week, 3 = 2‐3 times a week, 4 = most days, and 5 = everyday). Parents were also asked how many kinds of vegetables children ate, excluding potatoes but including vegetables in composite dishes like pasta and curry, this was used as a proxy of variety of consumption (the scale used was 0, 1‐4, 5‐9, and 10 or more kinds). To measure agency, parents were asked to report in the questionnaires if children had mentioned vegetables at home or if they engaged in vegetable related activities (e.g. play, stories, conversations about activities in nursery, asking to try/eat vegetables at home). The parental questionnaires were collected at the start and 2 weeks after the intervention. The questionnaires are available as Supporting material 2.

For process evaluation a log of activities was completed by the researcher during an observation conducted during week three in each nursery. In addition, observation forms were collected from nursery staff at the end of the study (week 4 and 2 weeks after completion), asking them to report activities they did during the intervention which were related to vegetable intake/exposure (Supporting material 3).

#### 2.4.2 Qualitative Outcomes

For qualitative outcomes, the open comment sections in the parental and nursery staff questionnaires were used. In addition, during the researcher observation in each nursery, engagement with the intervention was monitored, this was used to objectively report children's “agency.” During these observations short interviews with the nursery staff running the intervention were also conducted.

### 2.5 Data Analysis

IBM SPSS Version 29.0.1.0 was used for quantitative analysis. Vegetables tried were given one point per vegetable and summed for a score (maximum 27). The difference between post and pre scores were calculated to evaluate the change. Paired t‐tests were used to test statistical significance. Vegetable consumption was ranked on a scale of 1‐5 (1 = never/rarely, 2 = once a week, 3 = 2‐3 times a week, 4 = most days, 5 = every day). Variety of consumption was ranked and scored from 0 to 3 (0 = none, 1 = 1 to 4, 2 = 5‐9, 3 = 10 or more). Wilcoxon signed‐rank tests were used for categorical data to test the effect of intervention on frequency and variety of vegetables consumed. Chi‐square was used to analyse parent data on vegetables tried.

The log forms used for process evaluation from observations by the researcher and nursery staff were tabulated. The RE‐AIM evaluation framework was used to report process evaluation (Glasgow et al. 2019)

For qualitative analysis a thematic analysis was used as described by Braun and Clarke (Braun and Clarke 2006). Information for qualitative analysis was extracted from open‐ended comments from the questionnaires for parents and nursery staff as well as from observer's notes. Eighteen out of 34 parents in the intervention group responded with comments discussing engagement in the home while only 2 out of 23 control group parents had comments. Thus, due to the lack of intervention in the control nurseries, the comments sections were not completed and not included in the qualitative analysis. All data was transcribed using NVIVO version 14.23.0. The researcher read and reread the data and noted key words that were used for coding. These were then used for the analysis resulting in three themes. Text was coded for words indicating whether vegetables were brought home to be used, vegetable related activities that took place in the nursery and conversation around vegetables: words related to touch, taste, smell, playing (imaginative), cooking, shopping, and prepping vegetables; and words indicating positive, negative, and neutral views about the Yes to Veg! Programme (Braun and Clarke 2006).

## 3 Results

### 3.1 Quantitative Outcomes

**Figure 1:**
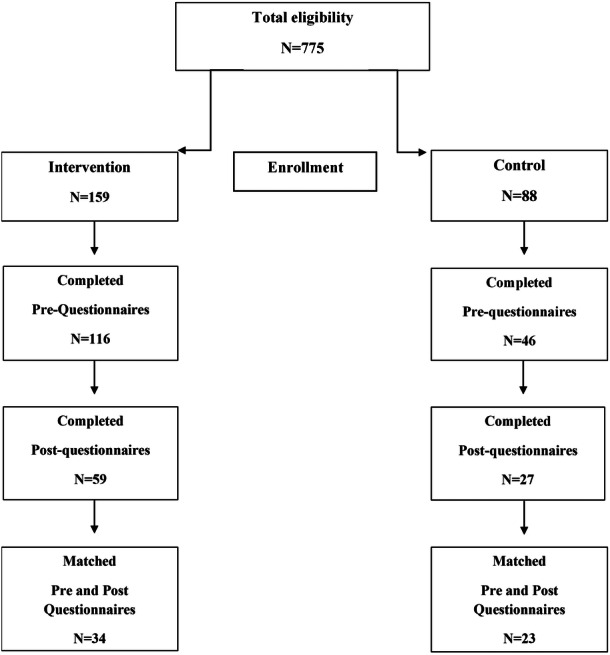
Participant recruitment and questionnaire completion flow.

One nursery from the control group dropped out of study at the start due to limited staff capacity to engage with the research, thus 11 nurseries (6 intervention and 5 controls) provided consent and were recruited for the study. A total of 257 parent‐child dyads were recruited, from this 162 completed pre questionnaires and 86 completed post questionnaires. A total of 57 parent dyads completed the two assessment points and were included in the analysis (Figure 1). The majority of children lived in households belonging to SIMD 1 or 2, classified as most deprived (65%) or deprived (21%). The age of children ranged between 35 and 67 months (mean age was 51 months) and 52% were boys. The parent reporting for children was mostly the mother (84%), there were 9% of children with learning support needs and 26% spoke another language different from English at home. The characteristics of participants in intervention and control groups were similar (Table 1).

**Table 1:** Sociodemographic information on the study population.

	Intervention		Control		Total		
	*n*	%	*n*	%	*n*	%	Chi square^a^
Sex^b^							0.861
Boy	18	52.9	12	52.2	30	53.6	
Girl	15	44.1	11	47.8	26	46.4	
Learning needs							0.80
Yes	3	8.8	6	26.1	9	15.8	
No	31	91.2	17	73.9	48	84.2	
Other language							0.031
Yes	9	26.5	1	4.3	10	17.5	
No	25	73.5	22	95.7	47	82.5	
SIMD^b^							0.075
1 = Most deprived	24	70.6	13	56.5	37	67.3	
2 = Deprived	7	20.6	5	21.7	12	21.8	
3, 4, 5 = Less to no deprived	3	8.8	3	13	6	11	
Parent relationship							0.208
Mother	30	88.2	18	78.3	48	84.2	
Father	4	11.8	3	13	7	12.3	
Other	0	0	2	8.7	2	3.5	

^a^ Chi square for comparisons between intervention and control.

^b^ *n* = 2 missing SIMD information from control group, *n* = 1 missing sex info from intervention group.

Exposure outcomes included individual vegetables tried before and after the intervention and mean scores (SD) with differences between pre and post measurements in intervention and control. There were no statistical significant differences in the total scores for vegetable tried between intervention [total score pre 16.7 (4.5 SD) vs 16.8 (5.6 SD) post, difference 0.19 (0.66 SD) *p* = 0.765] and control groups [total score pre 16.4 (5.3 SD) vs 16.0 (5.6 SD) difference −0.39 (0.57 SD), *p* = 0.503].

Consumption was reported as frequency and variety of vegetables consumed by children (Table 2). There were no changes in pre and post measurements for either outcome in both groups. The median consumption was 4 (most days); a trend in the frequency of consumption toward consuming every day was observed in the intervention group (*p* = 0.059). The variety of vegetables consumed was different between intervention and control but there were no statistically significant changes between pre and post measurements. The median variety of vegetables consumed in the intervention group was 2 (5–9 types of vegetables) this indicates that 50% could consume less than 5–9 kinds of vegetables whilst the control group reported a lower median variety of consumption of 1 (1–4 types of vegetables) (Table 3).

**Table 2:** Frequency of vegetable consumption.

	Intervention (*n* = 34)				Control (*n* = 23)			
	Pre		Post		Pre		Post	
Frequency	N	%	N	%	N	%	N	%
Everyday	5	14.7	7	20.6	3	13	5	21.7
Most days	14	41.2	16	47.1	11	47.8	10	43.5
2‐3 days/week	9	26.5	6	17.6	2	8.7	4	17.4
Once/week	1	2.9	3	8.8	4	17.4	1	4.3
Rarely/never	5	14.7	2	5.9	3	13	3	13
Median	4		4		4		4	
P25/75	4/4		3/4		2/4		3/4	
Z scores	−1.886				−1.613			
P‐value	0.059				0.107			

**Table 3:** Variety of vegetable consumption.

	Intervention (*n* = 34)				Control (*n* = 23)			
	Pre^a^		Post		Pre		Post	
Frequency	N	%	N	%	N	%	N	%
10 or More	6	17.6	7	20.6	7	30.4	6	26.1
5‐9	13	38.2	13	38.2	3	13	5	21.7
1‐4	12	35.3	14	41.2	12	52.2	11	47.8
None	2	5.9	0	0	1	4.3	1	4.3
Median	2		2		1			1
P25/75	1/2		1/2		1/3			1/3
Z scores	−0.832				0.00			
P‐value	0.405				1.00			

^a^ *n* = 33, one value missing.

At the end of the Yes to Veg! Programme 81% of parents reported that their children talked about vegetables at home; 39% indicated that their children had tried new vegetables and 30% indicated that their children had changed their eating habits. All staff from the Yes to Veg! Programme participating nurseries noticed a change in children's desire to try and engage with vegetables, this was not reported in the control nurseries.

### 3.2 Qualitative Outcomes

Three key themes were identified from parental, nursery staff and researcher observations.

#### 3.2.1 Theme 1—Engagement With the Intervention

Children gained agency during the intervention as reported by parents, nursery staff and confirmed via researcher observations. Agency was observed by children's engagement and participation with the topic of vegetables. According to parental reports, children talked about vegetables at home, they asked to eat vegetables in their meals at home. Nursery staff reported that children were talking about vegetables and were willing to try them. Agency could be mediated by active role‐playing during nursery. This was confirmed during researcher observations and interviews with nursery staff.

She is really enjoying telling us all about new veg.

(Parent 98)

Child has been asking for vegetables with her meals. This has never been the case. Usually, we need to use bribery to get her to taste veg.

(Parent 39)

Talked about vegetables more…. excitement.

(Nursery 2, Staff)

[staff]… they may not like the vegetable but were still willing to try it.

(Nursery 1 Staff)

I saw that cutting/prepping vegetable activities were among the most popular activities among nurseries and the children in the nurseries.

(Researcher)

Staff were excited to share stories of engagement with the intervention from both firsthand accounts and about ones that were read about in the parent portal related to the intervention.

(Researcher)

#### 3.2.2 Theme 2—Sensory Interaction

Access to vegetables during the intervention period facilitated children's sensory interaction. Parents' comments from what they experienced at home included:

Wanted to try some raw veg instead of it cooked.

(Parent 112)

Yes and no. He hasn't tried or eaten any veg at home but enjoyed chopping and touching it. It has been really good for getting him more aware of different veg; we will continue to chop veg to see if he will try some someday.

(Parent 50)

[staff]… mentioned that they have a child in their care who does not enjoy consuming vegetables and will refuse to have anything to do with them. However, one of her friends enjoyed vegetables a lot, and when they got the cucumber and tomatoes in for the intervention, the friend encouraged her to try a cucumber. Between the staff and her friend, they got the child to lick the cucumber which she stated ‘it was not bad’.

(Researcher)

…. there were lots of trying.

(Staff Nursery 2)

… children were licking vegetables.

(Researcher)

#### 3.2.3 Theme 3—Acceptance of Yes to Veg! Programme

Overall the views on the programme among parents and nursery staff were very positive.

It's a good initiative and supports the idea of children having access to different vegetables during mealtimes.

(Parent 80)

… it has been bringing new vocabulary to the children.

(Nursery 2 staff)

… enhancing play.

(Nursery 2 staff)

Nursery staff expressed that the intervention facilitated connections with parents

… nursery to home link, parents taking veg home.

(Nursery 5 staff)

… parent‐child‐interaction…. Shared experience on SeeSaw… [this] encouraging other families to participate.

(Nursery 5 staff)

The vegetable activities could be implemented alongside the existing activities in the nursery

The book “Supertato” *[by Paul Linnet and Sue Hendra]* is on our story of the month in the 2–3 room…relating to the tomatoes and cucumbers this week.

(Nursery 4 staff)

Nursery staff also expressed views on the delivery of the intervention

“…were disappointed when certain veg told to be delivered…and got different veg!”

However due to the funding and time available for the research, the engagement with the evaluation was limited, the study took place towards the end of the school year when there are several activities taking place and many children do not attend nursery as they normally would. The teachers were busier than usual and discussed this as a limitation of the process.

… not the best time of year for project…

(Nursery 1 staff)

### 3.3 Process Evaluation

Using the log of activities from researcher observations and the reports from nursery staff as well as findings from the qualitative analysis a more detailed account of process provides a better understanding of how the intervention was delivered (fidelity), who was reached and whether it was adopted by children, staff and parents. Table 4 shows the log of activities that were implemented and how the intervention was adopted in the Yes to Veg! Programme nurseries and in the control. All nurseries in the intervention implemented most of the suggested activities which shows high fidelity. In terms of reach, all children attending the Yes to Veg! nurseries received the intervention. With reference to adoption, observations by the researcher and reports from nursery staff on the types of vegetables that were popular with the children and with the parents (who were eager to take them home) indicated all nurseries had success with cucumbers, carrots, and tomatoes, followed by courgettes. Observations and interviews from the research were conducted during the third week of the intervention, so the staff could not speak about the success or failure of mushrooms and lettuce delivered during the fourth week of the intervention. Spinach and radish were less popular and often went to waste and parents were not keen to take them home. Maintenance was not monitored as part of the process as this was a 4‐week intervention only, however the comments from staff and parents were positive and they wished the intervention could be continued.

**Table 4:** Log of vegetable related activities observed and reported by nursery staff.

Table 4A. Activities observed by researcher in nurseries receiving Yes to Veg! Programme and in control nurseries.						
Yes to Veg! Nurseries						
Activity	Nursery 1	Nursery 2	Nursery 3	Nursery 4	Nursery 5	Nursery 6
Vegetable displayed	Y	Y	Y	Y	Y	Y
Vegetables served in meals	N	N	Y	N	N	Y
Playing with real vegetables	Y	Y	N	Y	Y	Y
Sensory activities using real vegetables	Y	Y	Y	Y	Y	Y
i‐ touching	Y	Y	Y	Y	Y	Y
ii‐ smelling	Y	Y	N	Y	Y	Y
iii ‐tasting/licking	Y	Y	Y	Y	Y	Y
Planting‐ growing vegetables	N	Y	N	Y	Y	Y
Role playing activities: selling/playing in shops	Y	Y	N	N	N	N
Food preparation with real vegetables eg cutting	Y	Y	Y	Y	Y	Y
Extra activities (with examples)	N	Y (naming vegetables)	Y (mud kitchen)	Y (mud kitchen)	Y (using carrots to roll pizza)	Y (mixing, mashing, transferring)

Control nurseries				
Activity	Nursery 1	Nursery 2	Nursery 3	Nursery 4
Vegetable displayed	N	N	N	N
Vegetables served in meals	N	Y	Y (potato, sweetcorn)	Y (always at lunch)
Playing with real vegetables	N	N (fake vegetable toys present)	N	N (fake vegetable toys present)
Sensory activities using real vegetables	N	N	N	N
i‐ touching	N	N	N	N
ii‐ smelling	N	N	N	N
iii ‐tasting	N	N	N	N
Planting‐ growing vegetables	N	N	N	N
Role playing activities: selling/playing in shops	N	N	N	N
Food preparation with real vegetables e.g. cutting	N	N	N	N
Extra activities	N	N	N	N

Table 4B. Activities reported by staff in nurseries receiving Yes to Veg! Programme and in control nurseries.						
Yes to Veg! Nurseries						
Activity	Nursery 1	Nursery 2	Nursery 3	Nursery 4	Nursery 5	Nursery 6
Are children engaging with play with vegetable voluntarily	Y	Y	Y	Y	Y	Y
Are there vegetables in the playroom	Y	Y	Y	Y	Y	Y
Are vegetable integrated into activities	Y	Y	Y	N	Y	Y
Are children playing with veg	Y	Y	Y	Y	Y	Y
Are children engaging with activities	Y	Y	Y	Y	Y	Y
Was it easy to fit vegetables into lessons	Y	Y	Y	Y	Y	Y
Did staff buy in to the intervention	Y	Y	Y	Y	Y	Y
Any noticeable changes in children's willingness to consume or play with vegetables	Y	Y	Y	Y	Y	Y
Can this be added to the curriculum	Y	Y	Y	Y	Y	Y
Did you have enough vegetables	Y	Y	Y	N	Y	Y
Did vegetable get taken home	Y	Y	Y	Y	Y	Y
List of activities	Baking	Vegetable printing	Role play	House corner	Measuring & comparing	Cooking
	Tasting	Counting	Cooking		Cutting & tasting	Planting
		Making patterns	Vegetable printing		Drawing	Bath/water play
		Hand‐ eye coordination activities	Still life observation & discussion		Planting	Story time
Most successful vegetables	Carrots	Carrots	Tomatoes	Carrots	Cucumber	Cucumber
	Cucumbers	Cucumbers	Courgette	Courgette	Tomatoes	Tomatoes
	Tomatoes		Cucumber	Radish		Courgette
			Carrots	Tomatoes		Carrot
				Cucumber		
				Spinach		

Control nurseries				
Activity	Nursery 1	Nursery 2	Nursery 3	Nursery 4
Are vegetables part of classroom conversations	Y (as part of meal times & planting)	Y (cooking, baking)	Y	Y (healthy eating, making soups, smoothies)
Are there current activities that use vegetables	Y (making snacks, painting)	Y (cooking, baking)	Y (try to cook & eat veg)	Y (cooking, printing, growing)
Does Eeatwell guide aid conversations around vegetable intake	N	Y	Y	Y (talk around 5 a day)
Do the children know what the Eatwell Guide is	N	N	N	N
Since the Eatwell Guide was sent home have you had more conversations about vegetables	N	N	Y	N
During mealtime do children eat vegetables provided	Y	Y (limited variety)	Y	Y
If yes, what are they most likely to eat	Onion	Peas	Broccoli	Carrot
	Peas	Sweetcorn	Sweetcorn	Onion
	Sweetcorn Cucumber Carrot	Carrots/cauliflower/broccoli medley	Carrots	Turnips Peas Tomatoes
Is there a garden to grow vegetable	Y	Y	Y	Y
Would staff be keen to take part in the Yes to Veg! Programme	Y	Y	Y	Y

## 4 Discussion

This study aimed to evaluate an intervention aiming to improve the food environment to promote vegetable consumption in preschool‐aged children. The inclusion of physical changes to the food environment is considered a key component for the promotion of healthier food environments in early years (Hawkes et al. 2015) but there is limited impact evaluation on this matter.

The intervention showed limited quantitative outcomes but promising findings from parental, nursery and researcher qualitative observations. Particularly important were parental reports that children gained agency by talking about vegetables, indicating that their children tried new vegetables and participated in vegetable activities at home and in school.

A cross‐sectional study analysing preschool children's sensory play and fruit and vegetable consumption found that placing real fruits and vegetables in their play areas encourages trying these items (Coulthard and Sealy 2017). Yes to Veg! Programme placed real vegetables in the classrooms for the children to interact with. Our qualitative findings show that interactions with vegetables in the nursery had a positive impact on children becoming familiar with vegetables which permeated to the home environment as reported by parents.

We report contradicting impact on children's willingness to try vegetables, depending on the evaluation tool used, from the list of vegetables tried in the intervention group there was no change in the median scores after 4 weeks; in contrast, from qualitative reports parents indicated that their children were engaging in trying new vegetables. This highlights the value of including parental opinions to complement survey‐based findings but also puts a cautionary interpretation to the quantitative outcomes. This could have been because parents did not engage well with the questionnaires due to limited time or clarity in the research process.

Evidence from a meta‐analysis utilising RCTs, quasi‐experimental studies, and clustered controlled studies has reported that experiential learning was a beneficial way of promoting increased F&V consumption and healthy living (Dudley et al. 2015). However, we didn't observe increases in vegetable consumption in this study. This could have been due to the small sample size but also the duration of the intervention which might not have been long enough to achieve behaviour change. A systematic review of interventions aiming to improve F&V intake in nursery age children reported improvements in intake in studies where the exposure and duration of the intervention was long (e.g. repeated exposure minimum of 8–10 exposures and interventions lasting up to 3 months (Nekitsing et al. 2019).

Nevertheless, we observed that increased exposure to vegetables as part of the learning experience in nursery settings is beneficial as it contributes to familiarisation, and could in the long term encourage consumption. In addition, our process evaluation shows that parents and staff feedback was very positive. A clustered RCT testing the effect of learning and play with vegetables found that sensory play, regardless of the vegetables used, increased the likelihood of children's willingness to try new vegetables (Nekitsing et al. 2019). Previous research complements the theories that sensory play, repetitive play, and agency all aid in children's responses to vegetable intake in their diets (Caton et al. 2013). Furthermore, a systematic review of nutrition promotion activities in early year's setting showed that inclusion of a range of various strategies such as offering a variety of vegetables and providing small portions were associated with improvements in eating behaviours (Wang et al. 2022). Our study contributes to the body of evidence pointing out that early years settings are an important component of the environment that can contribute to a food system's approach for the promotion of healthy eating habits which can support behaviour change in the home environment (Matwiejczyk et al. 2018). Our findings show that sensory play and agency are great ways of introducing children to vegetables, as demonstrated by the increase in children consuming vegetables more frequently during the intervention.

Our process evaluation shows that the intervention was adopted well, that the fidelity to the activities suggested was high but also that the nursery staff used their own creativity to use vegetables in their daily activities. The Yes to Veg! Programme design reaches all children in the nursery which is important for universal access and reduction in diet inequalities. This intervention has great potential for further implementation as shown in this study the activities were successfully implemented.

This study's strength is the inclusion of a vulnerable group living in socio‐economically deprived neighbourhoods, gaining access to vulnerable populations is a complex process that can be achieved mainly throughout cross‐sectoral collaborations and partnerships (Bonevski et al. 2014). Another strength of the study is the experimental design by the inclusion of a control group whilst evaluating a real life intervention.

The limitations of the study include the size of the final sample included in the analysis. While this study did provide access to a population otherwise not accessible to the researchers, the number of matched responses was small. The sample size might have contributed to the lack of statistical effects on the outcomes of interest. Throughout the intervention, nursery staff relayed how it can be challenging to hear back from their parents, alluding to the low questionnaire response, parents with young children might find it challenging to be responsive to research due to the demands of early year's parenthood. Lack of participant engagement in socially disadvantaged groups is a common barrier to research implementation, and more attention should be paid to communicate and engage better with participants. Including parents in the co‐design of the intervention for future studies should be considered (Bonevski et al. 2014).

Another limitation of the study was the intervention design which does not include a randomisation component. This, however in the context of real‐life programmes becomes less relevant as we provide here findings that align better with what would happen in a real life situation.

The lessons learnt from this study show that coordination with the different parts of the food system is essential. Due to limited time for the evaluation component we could not engage well with parents to provide more face‐to‐face information on the study, and to explain the importance of completing both questionnaires. Coordinating a launch event to inform all the members of the food system could have been useful for raising awareness with parents.

Similarly, there were some issues with the provision of locally grown organic vegetables which limited access to the vegetables originally intended (one known and a less known). The intervention was to run over the course of the school week, Monday through Friday. However, vegetables were not delivered to nurseries until Tuesday afternoons, indicating vegetable intervention occurred mainly on Wednesdays through Fridays as nurseries tried to get parents to take home most of the vegetables over the weekend. Vegetable deliveries were planned for certain vegetables to allow staff members' time to plan activities for the vegetables; however, the local vegetable provider wanted to ensure the best quality vegetables were delivered and that they had enough of them for each nursery, so the vegetables delivered were sometimes switched not giving staff time to prepare for activities for the new vegetables.

For future implementation it is essential to coordinate with the nursery staff which time of year works best for them. The Yes to Veg! Programme ran between May and June which coincides with end of the school year. Teachers at multiple nurseries took annual leave, leaving the intervention to get passed from one staff member to the next. This made contact with staff difficult. The time a year will also bring an element of seasonality for available F&V, this can be used as an opportunity for children to learn about the changing seasons and to appreciate the importance of choosing F&V according to seasons which supports planetary health.

A further learning element is the role nursery staff play in the delivery and motivation during the research process; this can be a positive aspect but also in this quasi‐experimental study we noticed that the headteacher in one of the control nurseries was highly motivated. This resulted in more questionnaires being returned but also the activities that routinely take place in that specific nursery included healthy eating components which could have affected the results, this is a limitation in real life research. The control nurseries were not explicitly instructed to change any of their usual activities but they knew about the overall aim of the study.

The experiences and careful process documentation of the evaluation in this study can be used in future interventions aiming to adopt a whole system approach, this is feasible and can be delivered successfully in similar settings.

## 5 Conclusion

The Yes to Veg! Programme increased children's willingness to try new vegetables, exposure, engagement and familiarisation with vegetables and promoted agency in pre‐school aged children who received the intervention. The Yes to Veg! Programme was well implemented and received by staff, children and parents in the communities however modifying food intake was not achieved in the timeframe of the intervention pointing out the need of expanding the length and the reach of the intervention to parents.

## Author Contributions

A.L.G. conceived the study design, supervised data collection and analysis. Z.Z. collected data, undertook analyses, and produced the first draft of the paper. I.M. designed the intervention, supported recruitment and contributed to write up. A.P. contributed to study design and writing. All authors contributed to successive drafts and have approved the final draft.

## Ethics Statement

This study was conducted according to the guidelines laid down in the Declaration of Helsinki and all procedures involving research study participants were approved by the University of Glasgow College of Medical Veterinary and Life Sciences (MVLS) (Approval No: 200230273) and Glasgow's City Council Education Services (Reference number 23.53). Written informed consent was obtained from all participants.

## Conflicts of Interest

The authors declare no conflicts of interest.

## Supporting information

Supplemmentary Material 1 Yes to Veg! Programme Activity Suggestions for Nursery Staff.

Supplemmentary Material 2 Pre and Post Questionnaires.

Supplemmentary Material 3 Research Observation Log.

## Data Availability

The data that support the findings of this study are available from the corresponding author upon reasonable request.
