# Supplementary material for: Evaluation of the Yes to Veg! Programme, a Food Systems Approach to Increase Vegetable Exposure and Agency in Pre‐School Age Children: A Quasi‐Experimental Study
Source: Matern Child Nutr. 2025 Dec 4;22(1):e70145. doi: 10.1111/mcn.70145 (PMC12678838; doi:10.1111/mcn.70145)
Supplement: Supplementary file 1 — Supplemmentary Material 1 Yes to Veg! Programme Activity Suggestions for Nursery Staff. [file MCN-22-e70145-s003.docx]

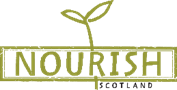

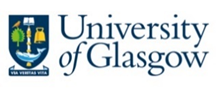


**Yes to Veg! Guide for Staff**

We are delighted your nursery will take part in the Yes to Veg! Programme. We want to make it easier for children under 5 to eat and enjoy vegetables in a fun and interactive way. This will be done by improving their food environment, making vegetables more accessible and helping them take an active role in the process.

**Background**

Research has shown us:

➢ Palates of young children are super sensitive, food fussiness can be common, and at this stage they form strong food preferences and eating habits for life. This is the perfect age to explore new flavours and textures. Embedding positive food experiences at this stage can impact powerfully on their longer-term health and well-being outcomes.

➢ Rather than passively being told, young children learn best through play, being on the move, and actively making their own choices.

➢ Young children respond well to repetition.

➢ Young children like to touch foods with their hands. They don’t like different foods mixed up.

**How will the project work?**

1. Link nurseries with local organic vegetable supplier.

2. Focus on 2 types of vegetables each week

3. Make vegetables a routine and normal part of the children’s surroundings

4. Embed vegetables in activities already happening at the nursery

5. Children take an active role in their food environment at nursery & at home

6. Share vegetables of the week with parents/carers to take home

We provide guidance and experience below about what worked well from our pilot projects. However, each nursery has the flexibility to adapt to fit their own situation using the 6 core themes above.

**1. Link nurseries with local Organic supplier**

Each participating nursery will be linked with a local organic supplier who will supply vegetables free of charge for the duration of the project. Connection with the people that produce our vegetables can help young children understand where our vegetables come from

The children could take turns to welcome the delivery of the vegetables, meeting the local supplier and be encouraged to be curious - for example, they could ask: “*Where did the vegetables come from*?” “*How far did it travel*?” “*What time of year does it grow best*?” “*How is it delivered?”* For example, Locavore uses electric vans. Children could also help staff carry the vegetables to the kitchen, help to wash and cut them and share them with their peers.

**2. Focus on 2 types of vegetables per week**

Young children learn well with repetition. By focussing on only 2 types of vegetables per week with lots of repetition throughout the week, children are likely to become used to and comfortable with the vegetables being used.

**3. Make vegetables a routine and normal part of the children’s surroundings**

Repetition & play is key. Sensory learning allows young children to learn by using their senses (taste, hearing, sight, smell, touch) and play freely. This can be done by displaying the vegetables in all areas of the nursery, so children could play with them and help themselves to the vegetables whenever they want to.

▪ Using real vegetables in the shopping corner is more fun

▪ Taking the seeds from the tomatoes could be messy but they can dry the seeds and try to grow their own tomatoes. The texture of squishy tomatoes can be an interesting sensation

▪ Rubbing kale leaves makes a funny noise

▪ Do you know you can turn a carrot into a recorder?

▪ Onions are smelly and can make you cry – why is that? And why do people cry sometimes?

▪ Broccolis look like trees, let’s pretend they are the trees for the dinosaurs

▪ We can use beetroot as lipstick or to paint beautiful flowers

**4. Embed vegetables in activities already happening at the nursery**

Vegetables can help us with maths, for example by:

o Learning how to weigh them

o Measuring the length of vegetables

o Counting and subtracting

Explore songs about vegetables

Draw the vegetables of the week

By embedding vegetables in the activities already planned for the week we increase the repetition around vegetables being used that week.

**5. The children take an active role in their food environment at nursery & home**

We recommend encouraging the children to welcome the vegetables, help carry the vegetables, help wash and cut the vegetables and take turns to serve the vegetables to their peers. The vegetables should be displayed in different areas in the nursery. And where suitable, children should be able to help themselves to the vegetables when they want to.

**6. Share vegetables of the week with parents/carers to take home**

At the end of the day, parents and carers will also be invited to take the same vegetables home so they can share the experience as a family. All vegetables will be local, seasonal, and organic. In our pilot project:

➢ Some nurseries created “market stalls” at the entrance so parents can help themselves to the vegetables at pickup or drop-off times.

➢ Other nurseries provided bags of the vegetables and included a simple recipe that the children had been using in the nursery.

➢ Some gave small packets of seeds (these can be provided) for parents to grow at home with their children if they wanted to.

➢ Parents and carers were encouraged to share what they were doing at home either through feedback sheets, photos or social media. Examples of what other nurseries have done:

Crookfur Family Centre video - <https://www.youtube.com/watch?v=epfR5i_LDoo>

Treetop Family Nurture Centre – [Promoting veg consumption across the UK](https://www.youtube.com/watch?v=epfR5i_LDoo)

Many thanks for your support.

We look forward to the children having fun and getting to eat more vegetables at the same time. Contact details for further information Irina Martin, Senior Officer, Nourish Scotland (irina@nourishscotland.org.uk)
