# Supplementary material for: Evaluation of the Yes to Veg! Programme, a Food Systems Approach to Increase Vegetable Exposure and Agency in Pre‐School Age Children: A Quasi‐Experimental Study
Source: Matern Child Nutr. 2025 Dec 4;22(1):e70145. doi: 10.1111/mcn.70145 (PMC12678838; doi:10.1111/mcn.70145)
Supplement: Supplementary file 2 — Supplemmentary Material 2 Pre and Post Questionnaires. [file MCN-22-e70145-s002.docx]

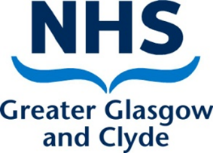

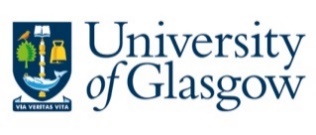

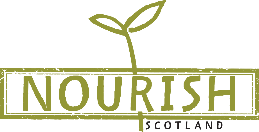


**Yes to Veg! Questionnaire (PRE)**

**1. I am the parent / grandparent / carer (please circle) of a:** Boy Girl Other

**2. What age is your child?** ______ years and ______ months

**4. Does your child have any additional support needs (please circle)?** Yes No

**If yes, what are these? __________________________________________________**

**__________________________________________________________________**

**5. Does your child speak another language at home (please circle)?** Yes No

**8. Which vegetables does your child most like to eat? (List all that you can think of. If you child doesn’t like to eat vegetables then write this down) ___________________________________**

**________________________________________________________________________________**

**9. Which of the vegetables in the list below have you AND your child tried? (tick all that apply)**

|  | **You have tried** | **Your child has tried** |  |  | **You have tried** | **Your child has tried** |
| --- | --- | --- | --- | --- | --- | --- |
| Asparagus |  |  |  | Lettuce |  |  |
| Baked beans |  |  |  | Leeks |  |  |
| Beetroot |  |  |  | Mushrooms |  |  |
| Broccoli |  |  |  | Onions |  |  |
| Butternut squash |  |  |  | Okra |  |  |
| Cabbage |  |  |  | Peas |  |  |
| Carrots |  |  |  | Pak choi |  |  |
| Cauliflower |  |  |  | Red/green/yellow pepper |  |  |
| Celery |  |  |  | Sweetcorn |  |  |
| Courgette |  |  |  | Sweet potato |  |  |
| Cucumber |  |  |  | Spinach |  |  |
| Chard |  |  |  | Tomatoes |  |  |
| Green beans |  |  |  | Turnip |  |  |
| Kale |  |  |  |  |  |  |

**10. Are there any other vegetables your child has tried? (if yes please tell us which ones)**

**___________________________________________________________________**

**___________________________________________________________________**

**11. How often does your child eat vegetables (not counting potatoes)? This includes vegetables in foods like pasta and curry)**

|  | **Every day** | **Most days** | **2-3 times per week** | **Once per week** | **Rarely or never** |
| --- | --- | --- | --- | --- | --- |
| **VEG (not potatoes)** | 🞎 | 🞎 | 🞎 | 🞎 | 🞎 |

**12. How many kinds of vegetables does your child eat? This includes vegetables in foods like pasta and curry)**

|  | **10 or more** | **5-9** | **1-4** | **None** |
| --- | --- | --- | --- | --- |
| **VEG (not potatoes)** | 🞎 | 🞎 | 🞎 | 🞎 |

**13. What are the 3 most common foods that your child eats as a snack between meals?**

­­­­­­­ 1. ________________________ 2. _____________________ 3. ______________________

**14. How often do YOU eat veg (not counting potatoes)? (This includes vegetables in foods like**  **pasta and curry)**

|  | **Every day** | **Most days** | **2-3 times per week** | **Once per week** | **Rarely or never** |
| --- | --- | --- | --- | --- | --- |
| **VEG (not potatoes)** | 🞎 | 🞎 | 🞎 | 🞎 | 🞎 |

**15. How many different kinds of vegetables do YOU eat? This includes vegetables in foods like**  **pasta and curry)**

|  | **10 or more** | **5-9** | **1-4** | **None** |
| --- | --- | --- | --- | --- |
| **VEG (not potatoes)** | 🞎 | 🞎 | 🞎 | 🞎 |

**Now a few questions about yourself and your family:**

**1. What relation are you to the child? (please tick)**

🞎 Mother 🞎 Father

🞎 Other (specify): **______________________**

**2. What is your postcode? ____________________**

**3. What age are you? _____________ years**

Thank you for completing this form 😊


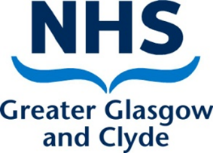

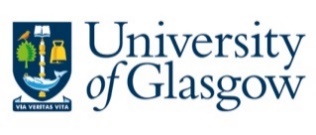

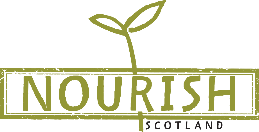


**Yes to Veg! Questionnaire (POST)**

**1. I am the parent / grandparent / carer (please circle) of a:** Boy Girl Other

**2. What age is your child?** ______ years and ______ months

**4. Does your child have any additional support needs (please circle)?** Yes No

**If yes, what are these? __________________________________________________**

**__________________________________________________________________**

**5. Does your child speak another language at home (please circle)?** Yes No

**8. Which vegetables does your child most like to eat? (List all that you can think of. If you child doesn’t like to eat vegetables then write this down) ___________________________________**

**________________________________________________________________________________**

**9. Which of the vegetables in the list below have you AND your child tried? (tick all that apply)**

|  | **You have tried** | **Your child has tried** |  |  | **You have tried** | **Your child has tried** |
| --- | --- | --- | --- | --- | --- | --- |
| Asparagus |  |  |  | Lettuce |  |  |
| Baked beans |  |  |  | Leeks |  |  |
| Beetroot |  |  |  | Mushrooms |  |  |
| Broccoli |  |  |  | Onions |  |  |
| Butternut squash |  |  |  | Okra |  |  |
| Cabbage |  |  |  | Peas |  |  |
| Carrots |  |  |  | Pak choi |  |  |
| Cauliflower |  |  |  | Red/green/yellow pepper |  |  |
| Celery |  |  |  | Sweetcorn |  |  |
| Courgette |  |  |  | Sweet potato |  |  |
| Cucumber |  |  |  | Spinach |  |  |
| Chard |  |  |  | Tomatoes |  |  |
| Green beans |  |  |  | Turnip |  |  |
| Kale |  |  |  |  |  |  |

**10. Are there any other vegetables your child has tried? (if yes please tell us which ones)**

**___________________________________________________________________**

**___________________________________________________________________**

**11. How often does your child eat vegetables (not counting potatoes)? This includes vegetables in foods like pasta and curry)**

|  | **Every day** | **Most days** | **2-3 times per week** | **Once per week** | **Rarely or never** |
| --- | --- | --- | --- | --- | --- |
| **VEG (not potatoes)** | 🞎 | 🞎 | 🞎 | 🞎 | 🞎 |

**12. How many kinds of vegetables does your child eat? This includes vegetables in foods like pasta and curry)**

|  | **10 or more** | **5-9** | **1-4** | **None** |
| --- | --- | --- | --- | --- |
| **VEG (not potatoes)** | 🞎 | 🞎 | 🞎 | 🞎 |

**13. What are the 3 most common foods that your child eats as a snack between meals?**

­­­­­­­ 1. ________________________ 2. _____________________ 3. ______________________

**14. How often do YOU eat veg (not counting potatoes)? (This includes vegetables in foods like**  **pasta and curry)**

|  | **Every day** | **Most days** | **2-3 times per week** | **Once per week** | **Rarely or never** |
| --- | --- | --- | --- | --- | --- |
| **VEG (not potatoes)** | 🞎 | 🞎 | 🞎 | 🞎 | 🞎 |

**15. How many different kinds of vegetables do YOU eat? This includes vegetables in foods like**  **pasta and curry)**

|  | **10 or more** | **5-9** | **1-4** | **None** |
| --- | --- | --- | --- | --- |
| **VEG (not potatoes)** | 🞎 | 🞎 | 🞎 | 🞎 |

**16. Did your child talk about vegetables in the past 4 weeks (please circle).**

Yes No

**17. Has your child asked to eat new vegetables over the past 4 weeks (please circle).**

Yes No

**18. Do you think Yes to Veg! has changed your child’s eating habits ? (please circle ).**

Yes No

**If yes, how? __________________________________________**

**19. Do you have any comments or feedback about Yes to Veg!**

**____________________________________________________________________________**

**____________________________________________________________________________**

Thank you for completing this form 😊
