# Supplementary material for: Evaluation of the Yes to Veg! Programme, a Food Systems Approach to Increase Vegetable Exposure and Agency in Pre‐School Age Children: A Quasi‐Experimental Study
Source: Matern Child Nutr. 2025 Dec 4;22(1):e70145. doi: 10.1111/mcn.70145 (PMC12678838; doi:10.1111/mcn.70145)
Supplement: Supplementary file 3 — Supplemmentary Material 3 Research Observation Log. [file MCN-22-e70145-s001.docx]

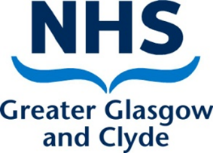

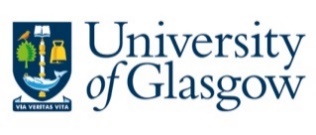

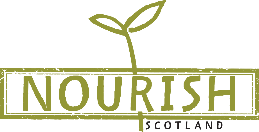


Nursery: ___________________________ Date: _______________

**Log of activities in Nursery**

**(Researcher Observation)**

**Availability**

|  | **Y/N** | **Comments, e.g. types, visibility,** |
| --- | --- | --- |
| **Vegetables displayed** |  |  |
| **Vegetables served in meals?** |  |  |

**Activities**

| **Activity in nursery** | **Yes / No** | **Number of children observed doing activities** | **Comments** |
| --- | --- | --- | --- |
| **Playing with real veg** |  |  |  |
| **Sensory activities using real veg**  **(List any activities observed)** |  |  |  |
| **Touching** |  |  |  |
| **Smelling** |  |  |  |
| **Tasting** |  |  |  |
|  |  |  |  |
| **Planting – growing veg** |  |  |  |
| **Role play activities; selling/playing in shops with real veg** |  |  |  |
| **Food preparation using real veg** |  |  |  |
| **Any other activities related to real veg – Please specify what activities** |  |  |  |

**Nursery staff engagement**

| **Type of activity/action** | **Comments, e.g. types, visibility,** |
| --- | --- |
|  |  |
|  |  |
|  |  |


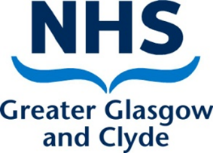

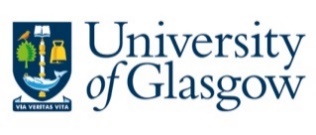

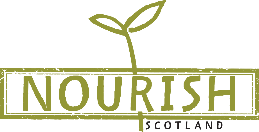


Nursery: ___________________________ Date: _______________

Member of staff who is completing the form, please write here your role (job title)

_____________________________________

**Log of activities in Nursery**

**(Nursery Staff)**

**Please complete this log of activities during week 5 by ticking which activities took place under**

| **Activity in nursery** | **Yes** | **No** |
| --- | --- | --- |
| **Play with real veg** |  |  |
| **Sensory activities using real veg (e.g. touching, smelling, tasting, listening to veg)** |  |  |
| **Planting – growing veg** |  |  |
| **Role play activities; selling/playing in shops with real veg** |  |  |
| **Food preparation using real veg** |  |  |
| **Parents took veg home** |  |  |
| **Any other activities related to real veg – Please specify what activities** |  |  |
